# Inhibition of MUC1 exerts cell-cycle arrest and telomerase suppression in glioblastoma cells

**DOI:** 10.1038/s41598-020-75457-z

**Published:** 2020-10-26

**Authors:** Sojin Kim, Youngbeom Seo, Tamrin Chowdhury, Hyeon Jong Yu, Chae Eun Lee, Kyung-Min Kim, Ho Kang, Hak Jae Kim, Soo-Ji Park, Kyoungmi Kim, Chul-Kee Park

**Affiliations:** 1 Department of Neurosurgery, Seoul National University College of Medicine, Seoul National University Hospital, 101 Daehak-ro, Jongno-gu, Seoul, 03080 Korea; 2 Department of Neurosurgery, Yeungnam University College of Medicine, Yeungnam University Hospital, Daegu, Republic of Korea; 3 Department of Radiation Oncology, Seoul National University College of Medicine, Seoul Nation University Hospital, Seoul, Republic of Korea; 4 Department of Biomedical Sciences and Department of Physiology, Korea University College of Medicine, Seoul, Republic of Korea

**Keywords:** Cancer, Molecular biology, Medical research

## Abstract

Mucin 1 (MUC1) is a transmembrane glycoprotein involved in tumorigenesis of diverse cancers. However, the role of MUC1 in glioblastoma (GBM) has not yet been fully explored. In this study, the anticancer mechanism of *MUC1* suppression in GBM was investigated. The expression level of *MUC1* was analyzed in human glioma and paired normal brain tissues. *MUC1* was overexpressed in GBM and was negatively associated with overall survival. Moreover, we silenced *MUC1* to investigate its effect in GBM cell lines and found that knockdown of *MUC1* inhibited cell proliferation and resulted in cell cycle arrest at G1 phase. *MUC1* silencing decreased the phosphorylation of *RB1* and increased the expression of *CDKN1B*. Gene set enrichment analysis showed that a series of genes related to cell cycle, telomere maintenance and transforming growth factor Beta (TGF-β) signaling in epithelial mesenchymal transition (EMT) were influenced by *MUC1* knockdown. Notably, the reduced *TERT* expression levels combined with impaired telomerase activity and the switching of telomere maintenance mechanism to alternative lengthening of telomeres (ALT) were observed after *MUC1* knockdown. Our results support the role of *MUC1* in oncological process in GBM which can be developed as a therapeutic target for cell cycle control and telomere maintenance mechanism.

## Introduction

Glioblastoma (GBM) is the most common primary malignant brain tumor in adults^1^. Despite standard treatments including surgery, radiotherapy and chemotherapy, the outcome of this malignant tumor remains dismal^1–3^. To date, several prognostic genetic/epigenetic biomarkers, such as isocitrate dehydrogenase (*IDH*) mutation, human telomerase reverse transcriptase (*hTERT*) promoter mutation, and O^6^-methylguanine DNA methyltransferase (*MGMT*) promoter methylation have been identified through extensive molecular and genetic studies for glioblastoma^4^. Among those biomarkers, *hTERT* promoter mutation is associated with expression of *hTERT* and elevation of telomerase activity (TA), which is one of the poor prognostic factor recently identified in gliomas^5^.

Mucin 1 (MUC1) is a single pass type I transmembrane protein with a heavily glycosylated extracellular domain^6,7^. Full length MUC1 compose with two subunits, N-terminal subunit (MUC1-N) and C-terminal subunit (MUC1-C). MUC1-C again consist of extracellular cellular domain (ECD), transmembrane domain (TMD), and cytoplasmic tail (CT)^7^. MUC1 is normally expressed at low levels on the glandular or luminal epithelial cells in breast, lung, gastrointestinal tract, pancreas, uterus and, prostate, and a lesser extent in hematopoietic cells^7,8^. However, aberrant glycosylation and overexpression of MUC1 has been described in most of human epithelial cancers and even in hematological malignancies^7,8^. Evidences showed that *MUC1* may act as an oncogene related to tumor formation and progression in many cancers^9–11^. Moreover, *MUC1* is reported to be associated with the cancer invasiveness and metastasis, neo-angiogenesis, drug resistance, and poor prognosis^12–15^. Thus, *MUC1* is thought to be the universal player that acts in various steps of oncogenesis.

Epithelial-mesenchymal transition (EMT) is well known oncogenic process accelerating invasiveness and metastasis of cancer cells^16^. MUC1 CT upregulates the EMT inducers directly as well as indirectly by modulating the expression of miRNAs that control gene expression related to EMT^11,17^. Transforming Growth Factor Beta (TGF-β) is a cytokine with a dichotomous role in tumorigenesis^18^. TGF-β1 plays a role as a tumor suppressor which induces apoptosis or cell suicide by SMAD signaling pathway at early stage of oncogenesis^19,20^. However, in the late stage of aggressive and invasive tumors, TGF-β signaling stimulate tumor progression by its pleiotropic activities on the cancer cells which include induction of EMT, migration, invasion, and tumor metastasis^18,21,22^. *MUC1* is reported as a key inducer of EMT and is partially responsible for the functional switch of TGF-β from a tumor suppressor to a tumor promoter during EMT in multiple cancers^23^. However, the role of *MUC1* has not been fully elucidated in GBM, although only sporadic reports mentioned the *MUC1* in relation to the maintenance of aggressive of gliomas^24^.

In this study, we examined the anticancer effect of *MUC1* suppression in GBM. Our work reveals a role of *MUC1* in GBM oncogenesis involving cell cycle control and telomere maintenance mechanism, which can be developed as a potential prognostic marker and therapeutic target in GBM.

## Results

### *MUC1* is significantly overexpressed in GBM tissue

**Figure 1:**
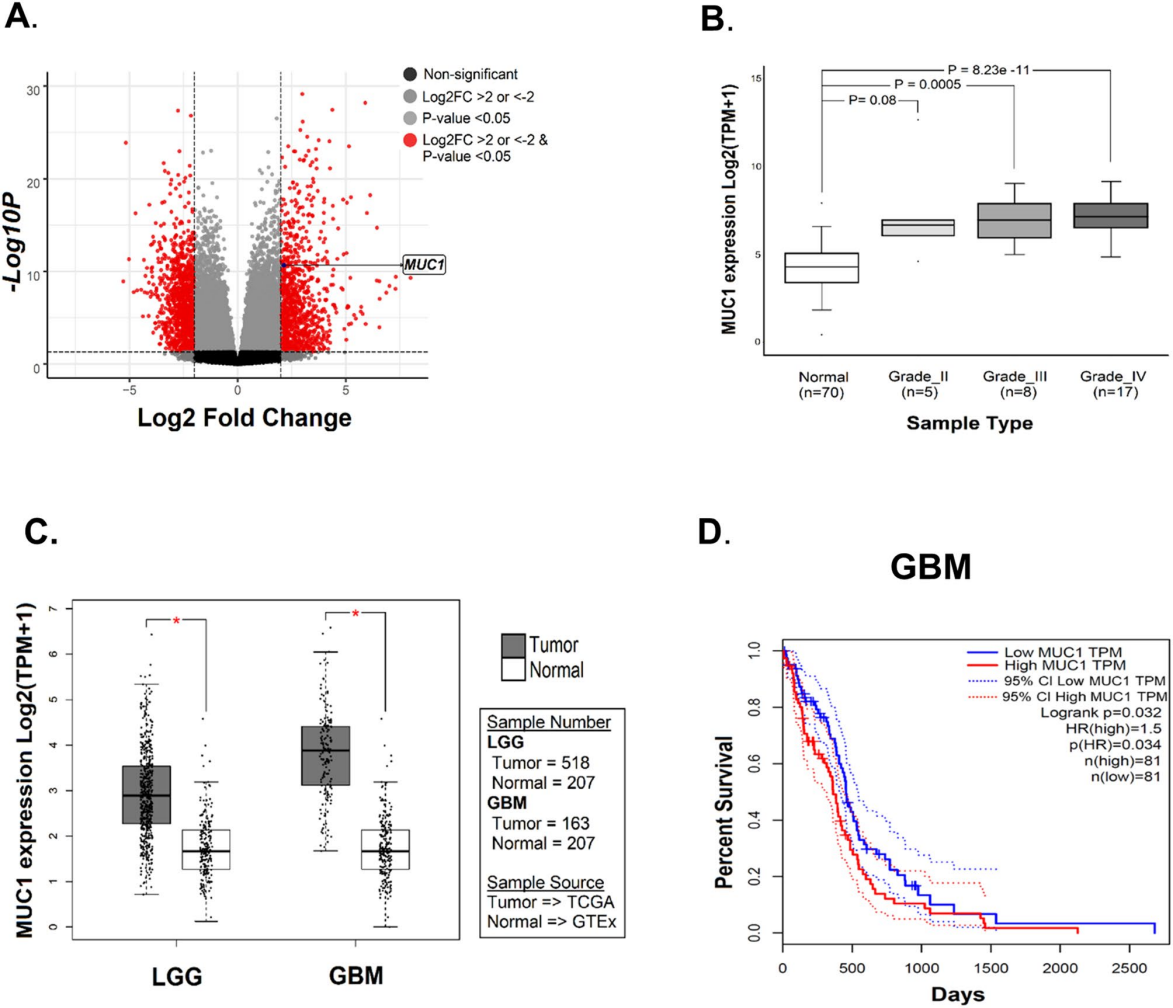
*MUC1* is significantly overexpressed in gliomas. (**A**) Identification of differentially expressed genes from the RNA-seq data of paired normal brain and glioma tissue of 30 glioma patients. Volcano plot showing *MUC1* as one of the significant upregulated gene. (**B**) Comparison of *MUC1* mRNA expression in normal brain tissue and glioma (WHO grade II, III, and IV) tissues. *MUC1* is significantly overexpressed in all glioma samples compared to normal brain samples. (**C**) Comparison of *MUC1* RNA expression in glioblastoma (GBM) and lower grade glioma (LGG) data from TCGA (The Cancer Genome Atlas) with normal brain data from GTEx (Genotype-Tissue Expression). *MUC1* is highly expressed in both GBM and LGG. (**D**) Overall survivals in GBM patients in TCGA dataset classified by *MUC1* expression. The prognosis is significantly poor in *MUC1* overexpressed patients.

In order to study the mechanisms of differential gene expression in glioma tumorigenesis, we performed gene expression profiling by RNA sequencing data of paired normal brain and glioma tissue of 30 glioma patients. Among the differentially expressed genes identified, *MUC1* was one of the significantly upregulated genes (p-value < 0.05, log2FC ≥ 2) in glioma tissue (Fig. 1A). However, the role of *MUC1* in glioma cells has been rarely studied. The upregulation of *MUC1* was a universal phenomenon in gliomas regardless of their WHO grades, although only high-grade gliomas including GBM showed statistical significances (Fig. 1B).

Using GBM and lower grade glioma (LGG) data from TCGA (The Cancer Genome Atlas) and normal brain data from GTEx (Genotype-Tissue Expression), we could confirm that the *MUC1* was overexpressed in glioma tissue compared with normal brain (Fig. 1C). Moreover, there was significant difference in overall survival between the groups of low- and high-expression level of *MUC1* in TCGA dataset of GBM (Fig. 1D).

### Knockdown of *MUC1* inhibits the proliferation of GBM cells

**Figure 2:**
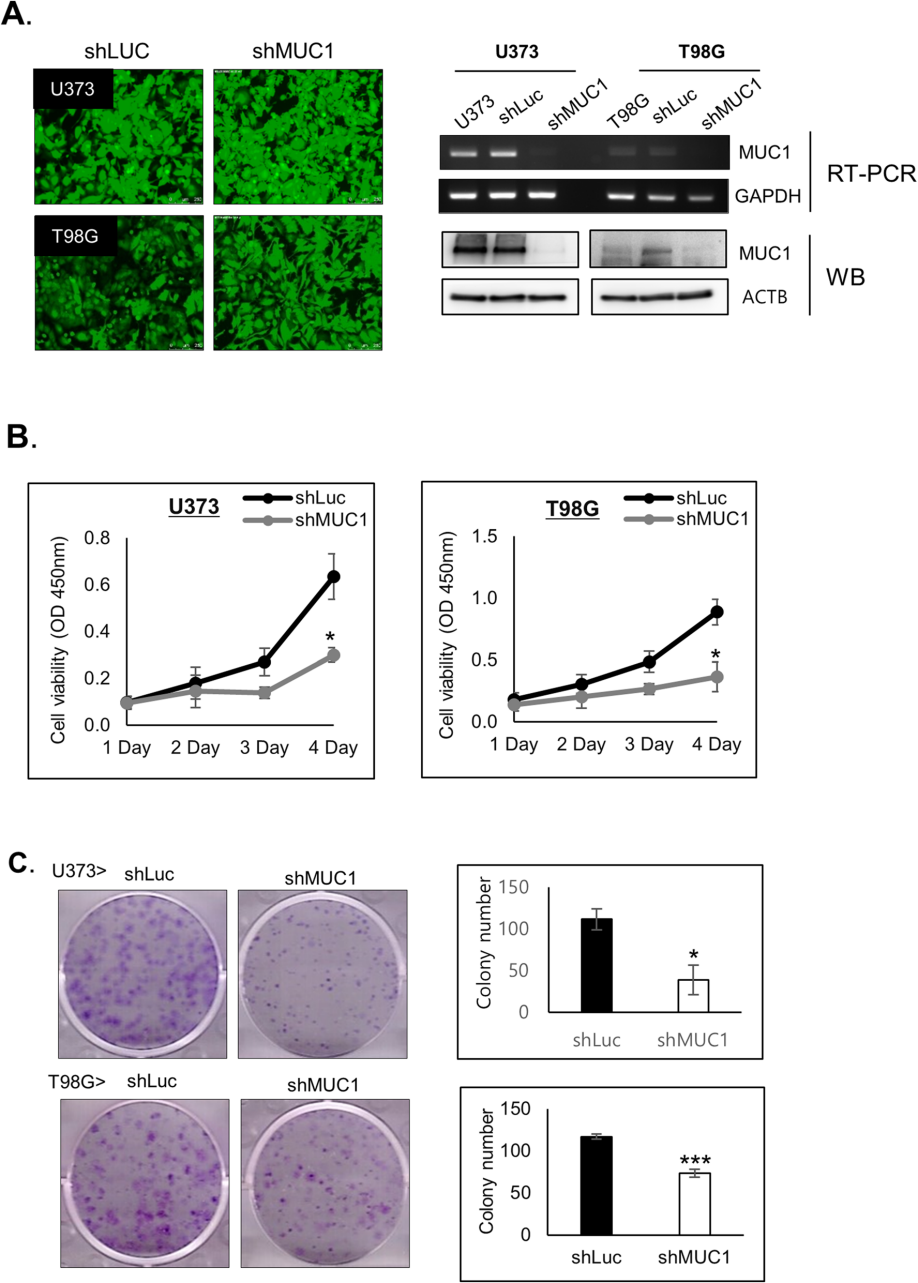
*MUC1* Knockdown inhibits the proliferation of glioblastoma cells. (**A**) GFP fluorescence images (magnification, × 100), RT-PCR and western blot (WB) analysis to confirm *MUC1* depletion in U373 and T98G with shLuc or sh*MUC1* lentivirus infection. GADPH and ACTB were used as an internal control in RT-PCR and western blot, respectively. The blots were cropped and full length blots are presented in Supplementary Fig. S1. (**B**) Cell proliferation assay for 4 days and (**C**) Colony-formation assays with T98G and U373 cells transduced with shLuc or sh*MUC1* lentivirus. After 14 days, the cultured cells were fixed with methanol and stained simultaneously with staining solution (0.5% crystal violet in acetic acid/methanol 1:7) and colonies were counted manually. (**B**,**C**) were representative results from at least 3 independent experiments and were plotted with average of 3 independent experiments. Error bars, SEM. Student’s t-test. *P < 0.05, **P < 0.01, ***P < 0.001.

To verify the role of *MUC1* in GBM cell growth, U373 and T98G cells were transduced with shLuc and sh*MUC1* lentivirus for 48 h and *MUC1* repression was confirmed with RT-PCR analysis and western blot analysis (Fig. 2A).

Cell viability test using MTT assay showed that *MUC1* knockdown decreased cell proliferation in U373 and T98G cells (Fig. 2B). Furthermore, Colony-formation assays also demonstrated reduced the size of the single colonies and the number of colonies in *MUC1* knockdown U373 and T98G cells (Fig. 2C). These results indicate that *MUC1* has a crucial role in GBM cell proliferation.

### *MUC1* knockdown attenuates cell cycle progression at G1 phase

**Figure 3:**
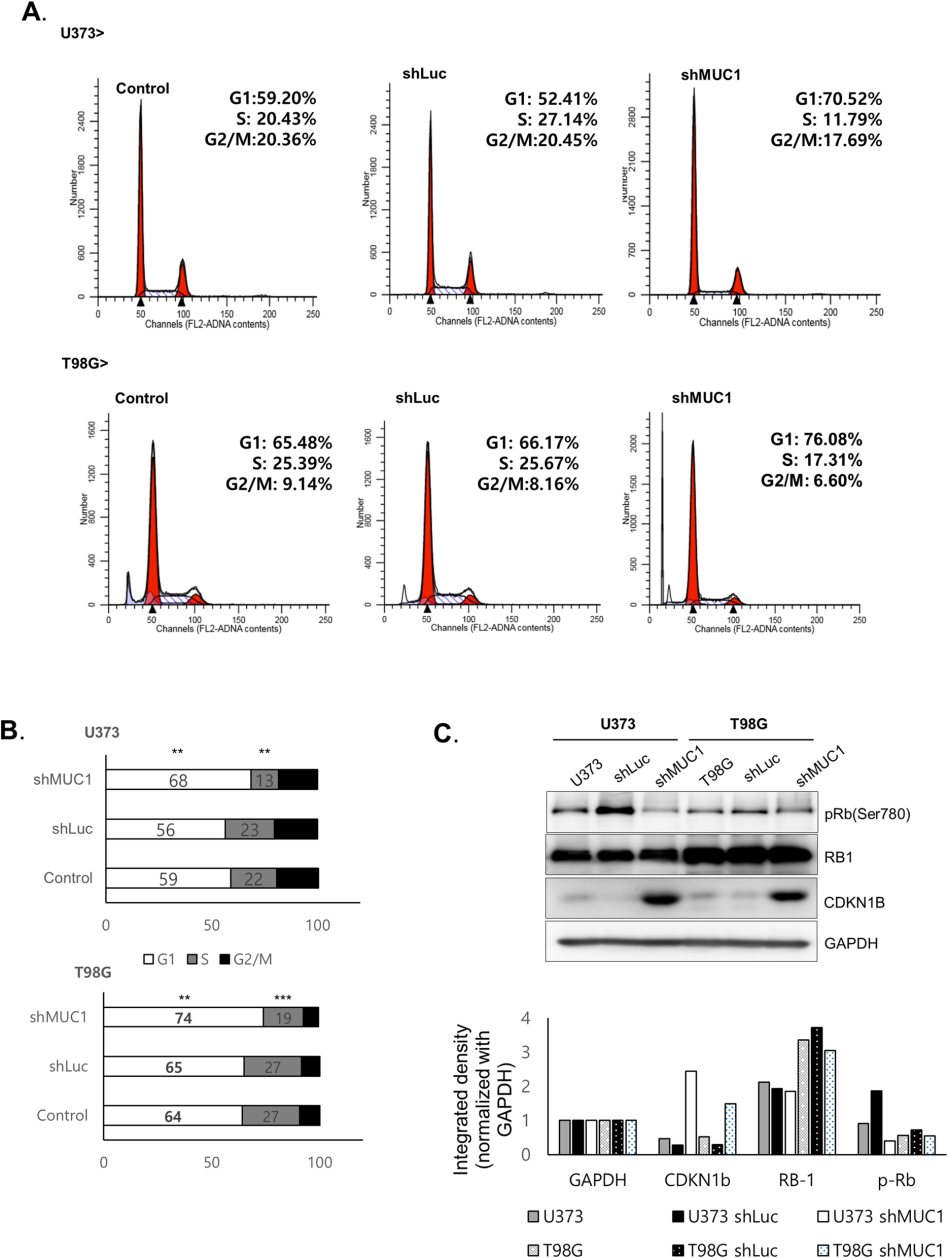
*MUC1* knockdown attenuates cell cycle progression at G1 phase. (**A**), (**B**) Cell cycle distribution of T98G and U373 with shLuc or sh*MUC1* lentivirus infection. In *MUC1* knockdown cell, cell cycle arrest in G0/G1 phase and reduction in S and G2/M phases were observed. Student’s t-test. *P < 0.05, **P < 0.01, ***P < 0.001. (**C**) Western blot analysis of Rb and CDKN1B in control and *MUC1* knockdown cells. The expression of GAPDH was used as an internal control. Target protein expression levels are were quantified by free image analyzer software. The blots were cropped and full length blots are presented in Supplementary Fig. S2.

To explore the underlying mechanism of decreased cell viability by *MUC1* knockdown, cell cycle analysis was performed. Flow cytometry showed that the cell cycle was stagnated in the G0/G1 phase after *MUC1* knockdown (Fig. 3A,B). Among the G1 cell cycle regulators, CDKN1B proteins were upregulated and phosphorylation of retinoblastoma 1 (*RB1*) was decreased upon *MUC1* knockdown (Fig. 3C). Control cells and *MUC1* knockdown cells were stained by annexin V and 7-AAD to see whether the G1 arrest is related with apoptosis. However, no statistical difference in apoptotic population was noted between control cells and *MUC1* knockdown cells (See Supplementary Fig. S4).

### Gene expression profiling reveals *MUC1*-related pathways other than cell cycle

**Figure 4:**
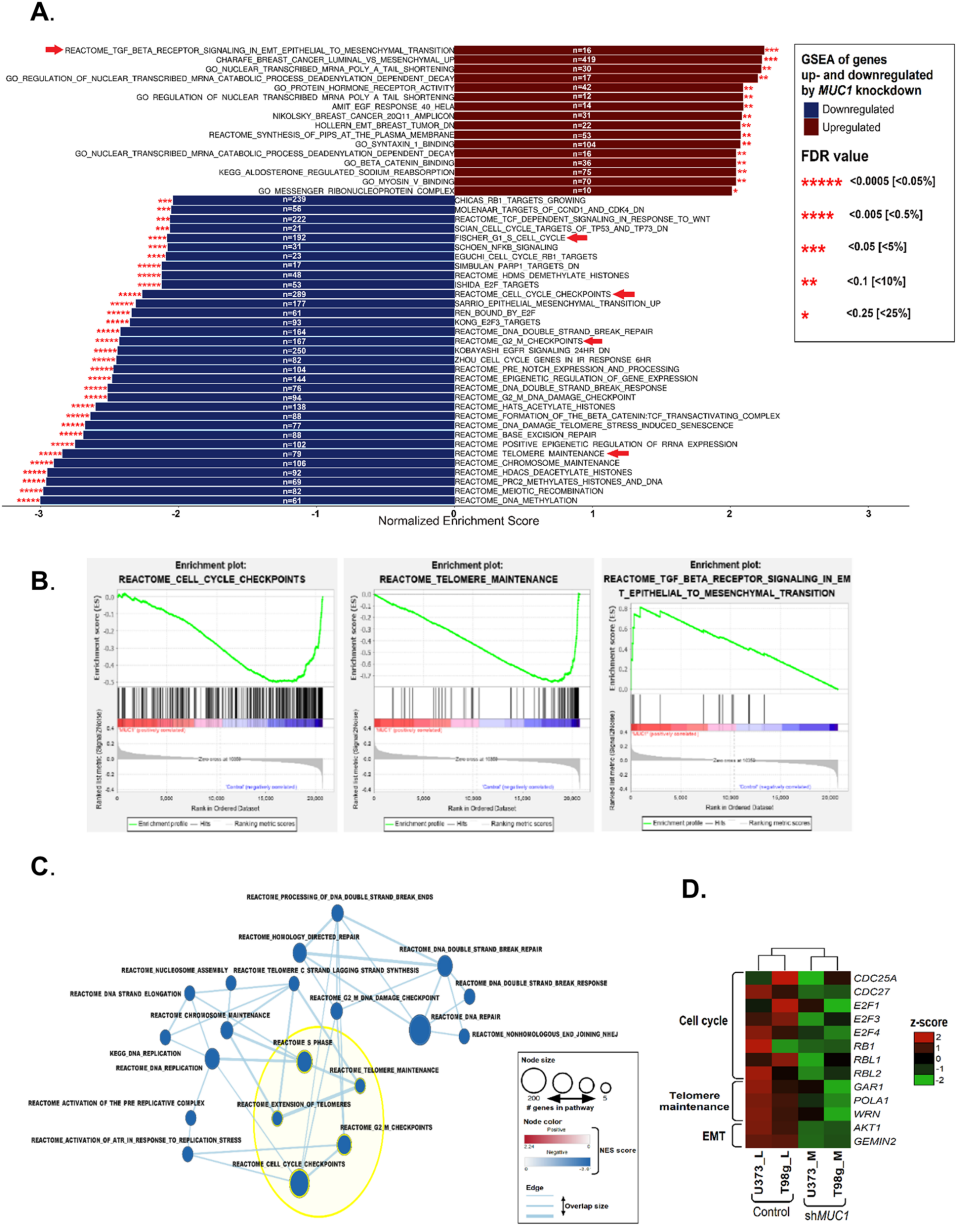
*MUC1* knockdown is associated with the cell cycle and telomere maintenance pathway. (**A**) Gene set enrichment analysis (GSEA) of control and *MUC1* knockdown glioblastoma (GBM) cell lines (T98G, U373, A172). TGF- β receptor signaling in epithelial mesenchymal transition (EMT) positively enriched in *MUC1* knockdown cell whereas gene sets associated with cell cycle and telomere maintenance pathway are negatively enriched. “N” indicates gene counts in enriched gene set. The asterisks indicate FDR q-values (*< 0.25; **< 0.1; ***< 0.05; ****< 0.005; ***** < 0.0005). Pathways with p-value < 0.05 and NES score of > 2 or < -2 are shown. (**B**) Examples of GSEA charts revealing the role of *MUC1* in GBM. Enrichment plot showing negative enrichment of cell cycle and telomere maintenance pathways and positive enrichment of TGF- β signaling in EMT pathway between control and *MUC1* knockdown cell lines. (**C**) Enrichment map was drawn using Cytoscape with the GSEA result (C5 gene set from MSigDb), FDR cut off value 0.01, Edge cut off 0.5. Cell cycle pathways and telomere related pathways are interconnected together by common genes and all are negatively enriched in the *MUC1* knockdown cell lines. (**D**) Heatmap comparison of cell cycle, telomere maintenance and EMT related gene lists in control vs *MUC1* knockdown GBM cell lines.

To investigate the role of *MUC1* in molecular mechanism underlying the tumorigenesis of GBM cells, we have profiled transcriptomes of both naive- and sh*MUC1*-treated GBM cells (U373, T98G, and A172) by RNA-seq. Using differentially expressed gene analysis dataset, we performed gene set enrichment analysis (GSEA). After filtering based on significant nominal p values (p < 0.05) and NES score of ≥ 2 or ≤ -2, we found gene sets that were down-regulated by *MUC1* knockdown were enriched in processes related to the EMT pathway, cell cycle-related pathway and telomere-related pathway (Fig. 4A,B). On the other hand, gene sets that were up-regulated by *MUC1* knockdown were enriched in processes related to TGF-β signaling in the EMT pathway (Fig. 4A,B). Pathway network analysis using GSEA result showed telomere-related and cell cycle-related pathway gene sets associated with *MUC1* knockdown were indirectly interconnected with each other (Fig. 4C). In *MUC1* knockdown GBM cells, significantly down-regulated genes that regulate the G1 phase of cell cycle, telomere maintenance and EMT pathway are shown in the heatmap and hierarchical clustering analysis (Fig. 4D). Taken together, these results suggested the MUC1 promotes glioma tumorigenesis through cell cycle regulation, telomere maintenance mechanism and EMT.

### *MUC1* knockdown induces changes in telomere maintenance mechanism

The perpetual maintenance of telomere length is an essential characteristic of tumorigenesis, which mechanism achieved either by activation of telomerase or by alternative lengthening of telomeres (ALT). ALT is characterized by the presence of ALT-associated promyelocytic leukemia bodies (APB), extrachromosomal telomeric circular DNA (c-circle), heterogeneous telomere length, and increased telomeric recombination. Here we used C-circle assay and telomere length measurement to detect the ALT activity^25^.

**Figure 5:**
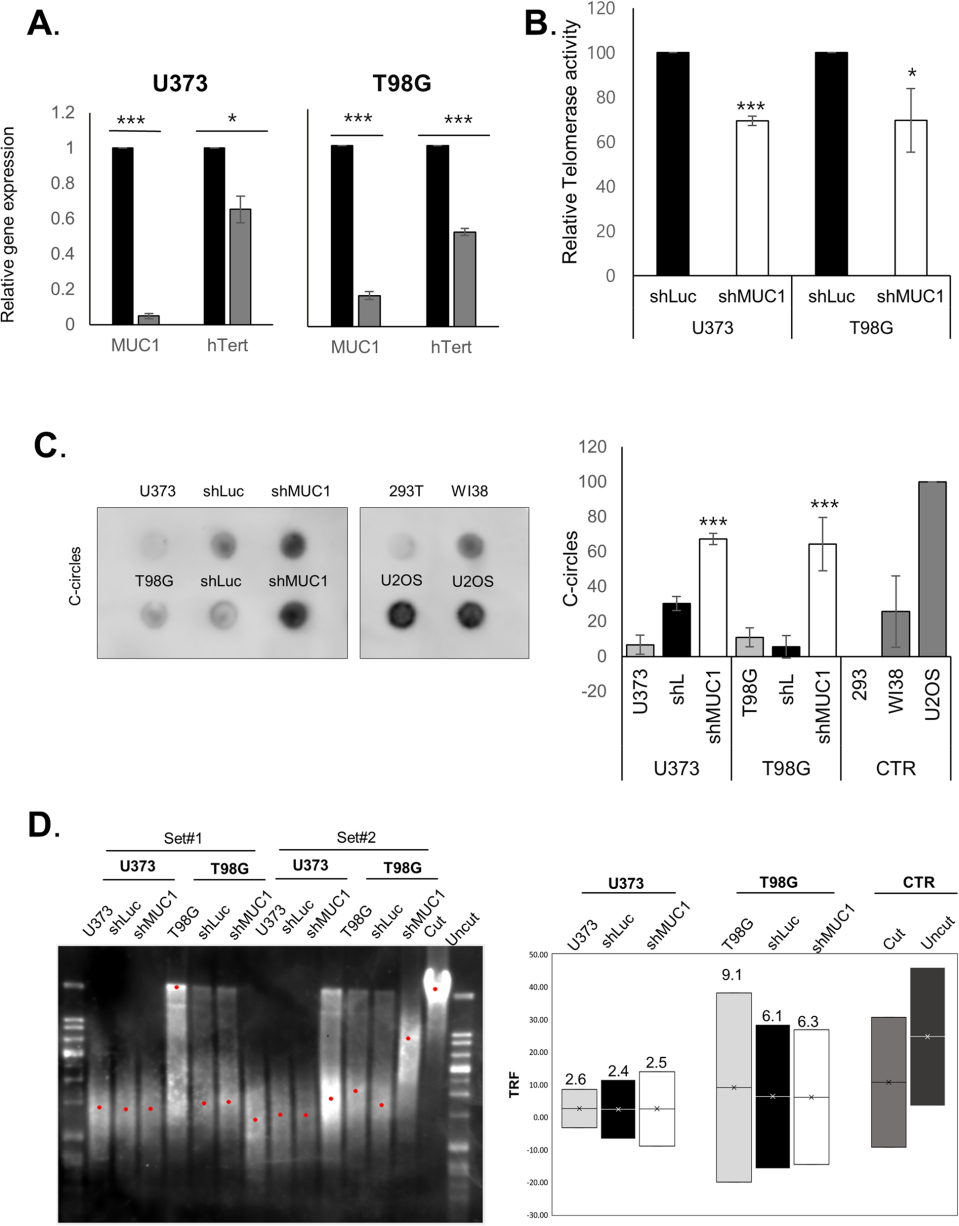
*MUC1* knockdown induces changes in telomere maintenance mechanism. (**A**) qRT-PCR results showing significantly lower *TERT* mRNA expression in *MUC1* knockdown cells. Data representative results from at least 3 independent experiments and were plotted with average of 3 independent experiments. Error bars, SEM. Student’s *t*-test. *P < 0.05, **P < 0.01, ***P < 0.001. (**B**) *MUC1* knockdown cells showing significantly reduce telomerase activity. Telomerase activities were analyzed using telomerase PCR ELISA kit and was quantified as ΔA (A450-A690) and then calculated as RTA (%). Data representative results from at least 3 independent experiments and were plotted with average of 3 independent experiments. Error bars, SEM. Student’s *t*-test. *P < 0.05, **P < 0.01, ***P < 0.001. (**C**) Blot showing positive formation of C-circles in *MUC1* knockdown glioblastoma (GBM) cells. Genomic DNA isolated from control (CTR) and *MUC1* knockdown T98G and U373 cells used for the C-circle assay. U2OS and WI-38 VA13 cells were used as the positive control and 293 T cell was used as the negative control. The blots were cropped and full length blots are presented in Supplementary Fig. S3. (**D**) Telomere restriction fragment (TRF) analysis using T98G and U373 cells infected with shLuc or sh*MUC1* lentivirus infection. Mean telomere restriction fragments (TRF) detected by southern blot analysis. Data are representative results from at least 3 independent experiments.

In order to validate the association of *MUC1* and telomere maintenance pathway, we tested *hTERT* expression and telomerase activity in GBM cells after *MUC1* knockdown, and we could observe significant reduction of *hTERT* expression as well as telomerase activity (Fig. 5A,B). We also performed the c-circle assay to elucidate whether *MUC1* knockdown affects the switch of telomere maintenance mechanism. *MUC1* knockdown induced a significantly increased c-circle formation (Fig. 5C). Additionally, telomere restriction fragment (TRF) analysis revealed telomere lengths were slightly increased in the *MUC1* knockdown GBM cells although there was no significant difference (Fig. 5D). Data are representative results from at least 3 independent experiments. Taken together, these results suggested that *MUC1* depletion contributes to the switching of telomere maintenance mechanism from classic telomerase activation to ALT in GBM cells.

## Discussion

In this study, we explored a functional role of *MUC1* in tumorigenesis of GBM based on results from both in vitro experiments and genomic data of human samples. We observed that *MUC1* knockdown attenuated cell proliferation and impaired the cell cycle progression to S-phase. In fact, the role of *MUC1* as a cell cycle regulator and poor prognostic marker has been studied in various human cancer including breast cancer, pancreatic cancer and lung cancer via various mechanism including β-catenin, NF-κB, platelet-derived growth factor (PDGF), epidermal growth factor receptor (EGFR), and MAPK and PI3K/Akt pathways^11,15,26,27^. However, the functional role of *MUC1* in GBM has not been clearly clarified so far. As mentioned above, the inhibition of cell proliferation and cell cycle arrest induced by *MUC1* knockdown, as detected by in vitro experiments in this study, suggest an oncogenic role of *MUC1* in GBM. More specifically, our work shows that the cell cycle was stagnated in the G0/G1 phase after *MUC1* knockdown involving *CDKN1B* up-regulation and decreased phosphorylation of *RB1*. The *CDKN1B* gene encodes for the p27Kip1 protein, which firstly described as an inhibitor of G1 cycle progression by binding a broad range of cyclin-CDK (cyclin-dependent kinase) complexes^28^. Moreover, *RB1* plays a central role in G1-S checkpoint control and phosphorylation of *RB1* is the most common mechanism of inactivation of this gene^29^. We found that *MUC1* Knockdown leads to increased *CDKN1B* expression and decreased phosphorylation of *RB1* in GBM cell lines. Taken together, these results suggested the effects of *MUC1* in cell cycle regulation, especially at the G1 phase in GBM.

Transcriptome profiling revealed large numbers of gene sets that are up- or down-regulated by *MUC1* knockdown. GSEA for the biological function of the genes up-regulated by *MUC1* knockdown identifies functions related to TGF-β signaling in EMT pathway. Previous study shows that TGF-β induces G1 growth arrest and the accumulation of unphosphorylated Rb^30^. These reports support our finding of role of MUC1 as a G1 phase regulator of cell cycle as shown in Fig. 3. The mechanism how *MUC1* gene silencing induced TGF-β signaling require further study. On the other hand, GSEA of genes down-regulated by *MUC1* knockdown identifies cell cycle-related pathway, EMT pathway, and telomere-related pathway. This result supports the experimental result showing cell cycle arrest after decreased *MUC1* expression. Telomere-related pathway, another suppressed pathway after *MUC1* knockdown, is one of the core elements in tumorigenesis. It has been proposed that up to 90% of the human cancers utilize telomerase activation for the maintenance of their telomere length, while the other 10% of tumors utilize ALT^31^. As has been reported in other cancers including gliomas, there is a correlation between telomerase activity with the grade of malignancy and prognosis in glial tumors^32–34^. However, others suggested that telomerase-associated parameters might have limited value as independent prognostic markers in a younger GBM patient^34^. Recent study by Hu et al. showed a possibility of switching phenomenon of telomerase maintenance mechanism in cancer cells by inducing telomeric DNA damages and knockdown of *ATRX/DAXX* complex^35^. Interestingly, we could observe the similar switching phenomenon from telomerase-positive to ALT-positive cells after *MUC1* knockdown. The identification of the role of *MUC1* in playing as a regulator for telomere maintenance mechanism is a novel discovery. A further study is needed to develop the detailed understanding of this switching mechanism of telomerase maintenance associated by MUC1.

We investigated the role of *MUC1*, a gene which was one of the widely studied in cancer except for GBM, focusing on its anti-cancer mechanism by inhibition. Our research suggested the role of *MUC1* as a regulator of cell cycle and telomere maintenance mechanism in GBM. It is expected that abrogating MUC1 can be considered as one of the therapeutic strategies for GBM.

## Materials and methods

### Cell culture

Human glioblastoma cell lines T98G, U373 and A172 were from Korean Cell Line Bank (Seoul, Republic of Korea) and the human embryonic kidney cell line 293 T was from American Type Culture Collection (ATCC). Cells were maintained in DMEM supplemented with 10% fetal bovine serum (J R Scientific), 100 U/ml penicillin and 100 μg /ml streptomycin sulfate (Welgene).

### Tumor specimens

We used prospectively collected samples of 30 histologically verified GBM patients who had undergone surgical resection. This study was performed under the approval of the Institutional Review Board of Seoul National University Hospital (IRB approval No., H-1608-139-787), and all experiments were performed in accordance with relevant guidelines and regulations. Written informed consent was obtained from all patients for the usage of samples.

### Gene knockdown

For gene silencing, shLuc and sh*MUC1* targeting CTTCGAAATGTCCGTTCGGTT of firefly luciferase gene and GACACAGTTCAATCAGTATA of human *MUC1*, respectively, were cloned into pLB lentiviral vector (generous gift from Dr. H. Y. Chung of Hanyang University). Lentivirus was produced by co-transfection of shLuc or sh*MUC1* with pMD2.G and psPAX2 into 293 T cells (ATCC) by calcium-phosphate method as described by the RNAi Consortium (2007). T98G and U373 cells were infected with lentiviral supernatant with 8 μg /ml protamine sulfate for 10 h. Gene knockdown was examined 48 h post infection^36^.

### RNA sequencing and analysis

Total RNA was isolated from GBM tissues using RNeasy Lipid Tissue Mini Kit (Qiagen) and the compatible library was prepared using the TruSeq stranded total RNA LT sample prep kit (Illumina, San Diego, CA, USA) according to the instructions specified by the manufacturer. Sequencing was done using NovaSeq 6000 system (Illumina). RNA expression levels were estimated using HISAT2 (version 2.1.0). The reference genome sequence (hg19, Genome Reference Consortium GRCh37) and annotation data were downloaded from the UCSC website (https://genome.uscs.edu). The transcript counts in gene level, and the relative transcript abundances in FPKM (fragments per kilobase of exon per million fragments mapped) were calculated using StringTie (version 1.3.4d).

### Gene set enrichment analysis (GSEA) and pathway network construction

GSEA was carried out using the standard GSEA tool for Windows version 4.0.3 (https://www.gsea-msigdb.org/gsea/index.jsp) with 1000 gene set permutations. Normalized count of the genes were inputted. Gene sets used for the analysis were Hallmark, canonical pathways and the Gene Ontology (GO). The upregulated and downregulated pathways were then filtered by normalized enrichment score ≥ 2 or ≤ -2, nominal P value < 0.05, FDR q value ≤ 0.25. The results obtained from the GSEA were then used to create a network map of the pathways. Enrichment map app from the Cytoscape program (version 3.8.0) was used to create the pathway network.^37^.

### Cell proliferation assay

Cell proliferation assay was performed with EZ-Cytox (Daeillab Service) on cells initially plated at 1 × 10^3^ cells/well in 96-well plates and cultured for indicated times. The absorbance was measured using a microplate reader (Molecular Devices) at a wavelength of 450 nm.

### Colony-forming assay

The cells were seeded in 6-well plates at a density of 1000 cells/well incubated at 37 °C under an atmosphere of 5% CO_2_ for 14 days to grow colonies. After 14 days, the cell colonies were fixed and stained with 0.05% crystal violet–methanol–acetic acid solution. Plates with stained colonies were scanned and scored.

### Cell cycle analysis

Cells were stained with propidium iodide and analyzed by flow cytometry as described^38^. The distribution of the cell cycle was determined by flow cytometry with FACSCanto II flow cytometer and FACSDiva software (BD Biosciences).

### Annexin V apoptosis assay

Cells were stained with the Annexin V-APC and 7-aminoactinomycinD (7-AAD) (BD Biosciences) according to the manufacturer’s protocol. The apoptosis rate was then analyzed using a FACS Calibur flow cytometer (BD Biosciences) using Cell Quest Pro software (BD Biosciences).

### RT-PCR and quantitative PCR (qPCR)

Total RNA was isolated from the tissues using RNeasy Lipid Tissue Mini Kit (Qiagen) and cDNA was cDNA was synthesized using RNA to cDNA EcoDry Premix Oligo dT(Takara) according to the manufacturer’s protocol. Real-time quantitative PCR (qPCR) was performed using PowerSYBR Green PCR Master Mix (Applied biosystems). Primers used for RT-PCR reactions were as follows: TERT (forward, 5′-GGAGCAAGTTGCAAAGCATTG-3′; reverse, 5′-TCCCACGACGTAGTCCATGTT-3′), MUC1 (forward, 5′- TACCGATCGTAGCCCCTATG-3′; reverse, 5′-CTCACCAGCCCAAACAGG-3′), GAPDH (forward, 5′-TGGTCACCAGGGCTGCTT-3′; reverse, 5′-AGCTTCCCGTTCTCAGCCTT-3′).

### Western blot analysis

Western blot analysis was performed as described^39^. Antibodies against CDKN1B (sc-528), RB1 (sc-50) were from Santa Cruz Biotechnology, GAPDH (2118), phosphorylated RB1 at S780 (p-S780, 9307A), MUC1 (4538) and ACTB (4967) were from Cell Signaling Technology, HRP-conjugated IgGs (111-035-003 and 115-035-003) were from Jackson Immune Research. Immunoblots were visualized with ChemiDoc XRS system (Bio-Rad). The densities of bands were measured using free image analyzer software (ImageJ V1.8x; National Institutes of Health, USA, https://rsb.info.nih.gov/ij/).

### Telomere repeat amplification protocol (TRAP) assay with ELISA

The enzymatic activity of telomerase was measured using TeloTAGGG Telomerase PCR ELISA PLUS kit (Roche) according to the manufacturer’s protocol. GBM tissues and cells were homogenized in ice-cold lysis buffer using automill (Tokken). Briefly, after BCA protein quantification of the lysates, 10 µg of proteins were incubated in total volume of 50 µl reaction mixture at 25 °C for 30 min to allow the telomerase to add telomeric repeats to the end of the biotin-labeled primer. Consequently, PCR was conducted for 33 cycles of 94 °C for 30 s, 50 °C for 30 s, and 72 °C for 90 s, followed by additional extension time of 10 min at 72 °C and holding at 4 °C. The telomerase activity was measured at 450 nm and the reference wavelength 690 nm. Relative telomerase activity (RTA) of each sample was calculated according to the instruction of TeloTAGGG Telomerase PCR-ELISA PLUS Kit.

### C-circle assay

Detection of C-circles was performed as previously described^40^. Briefly, 30 ng DNA was combined with 10 µl 2X Φ29 Buffer, 7.5 U Φ29 DNA polymerase (NEB), 0.2 mg/ml BSA, 0.1% (v/v) Tween 20, 1 mM each dATP, dGTP and dTTP and incubated at 30 °C for 4 h and 8 h followed by 20 min at 70 °C. Amplification products were deposited on a Hybond N + nylon membrane (Bio-Rad) and developed using the TeloTAGGG Telomere Length Assay Kit (Roche). Chemiluminescent signals was visualized with ChemiDoc XRS system (Bio-Rad) and the intensity of the spots was quantified with ImageQuant TL software (Bio-Rad).

### Telomere length fragmentation assay

Telomere length was determined by southern blot using TeloTAGGG Telomere Length Assay Kit (Roche) according to the manufacturer’s protocol. Briefly, 1 µg DNA was digested with Rsa I and Hinf I for O/N at 37 °C, then electrophoresed on 0.8% agarose gel at 50 V for 4 h then transferred to a nylon membrane by Southern blotting. The blotting membrane was blocked and hybridized to a digoxigenin (DIG)-labeled probe specific for telomeric repeats for O/N. Washed blot was incubated with anti-DIG-alkaline phosphatase (1:10,00 dilution) for 30 min and developed using substrate in TeloTAGGG Telomere Length Assay kit (Roche). After Chemiluminescent signals was visualized with ChemiDoc XRS system (Bio-Rad), terminal restriction fragment analysis was performed with Telo Tool version 1.3.

### Statistical analysis

The results were analyzed by using IBM SPSS Statistics software (version 20.0; SPSS, Armonk, NY, USA). Data were expressed as the mean ± SE. Statistical significance was determined using the Student’s t-test. Kaplan–Meier curve analysis was used for analyzing patient survival time. P values < 0.05 were considered statistically significant.

## Supplementary information

Supplementary Information.
