# Supplementary material for: Inhibition of MUC1 exerts cell-cycle arrest and telomerase suppression in glioblastoma cells
Source: Sci Rep. 2020 Oct 26;10:18238. doi: 10.1038/s41598-020-75457-z (PMC7589558; doi:10.1038/s41598-020-75457-z)

**Title:** **Inhibition of MUC1 exerts cell-cycle arrest and telomerase suppression in glioblastoma cells**

Subtitle: Anticancer effect of *MUC1* inhibition in GBM

Sojin Kim^1*^, Youngbeom Seo^2*^, Tamrin Chowdhury^1^, Hyeon Jong Yu^1^, Chae Eun Lee^1^, Kyung-Min Kim^1^, Ho Kang^1^, Hak Jae Kim^3^, Soo-Ji Park^4^, Kyoungmi Kim^4^, Chul-Kee Park^1^,

^1^Department of Neurosurgery, Seoul National University College of Medicine, Seoul Nation University Hospital, Seoul, Republic of Korea

^2^Department of Neurosurgery, Yeungnam University College of Medicine, Yeungnam University Hospital, Daegu, Republic of Korea

^3^Department of Radiation Oncology, Seoul National University College of Medicine, Seoul Nation University Hospital, Seoul, Republic of Korea

^4^Department of Biomedical Sciences and Department of Physiology, Korea University College of Medicine, Seoul, Republic of Korea

* These authors contributed equally to this work.

**Corresponding author:**

Chul-Kee Park, MD, PhD.

Department of Neurosurgery, Seoul National University College of Medicine, Seoul National University Hospital, 101 Daehak-ro, Jongno-gu, Seoul 03080, Korea

Tel: 822-2072-0347, Fax: 822-741-8594, E-mail: [nsckpark@snu.ac.kr](mailto:nsckpark@snu.ac.kr)

**Supplements**

The full length blots for the gel images are given below in the pictures

Supplementary Figure 1.

Supplementary Figure 2.

Supplementary Figure 3.

Supplementary Figure 4.

**Supplementary Figure 1:** Full-length images of blots presented in figure 2A. Chemiluminescent signals were detected by exposure of the membranes in a Bio-Ras ChemiDoc system and the protein bands shown in Figure 2A are indicated by dotted boxes.


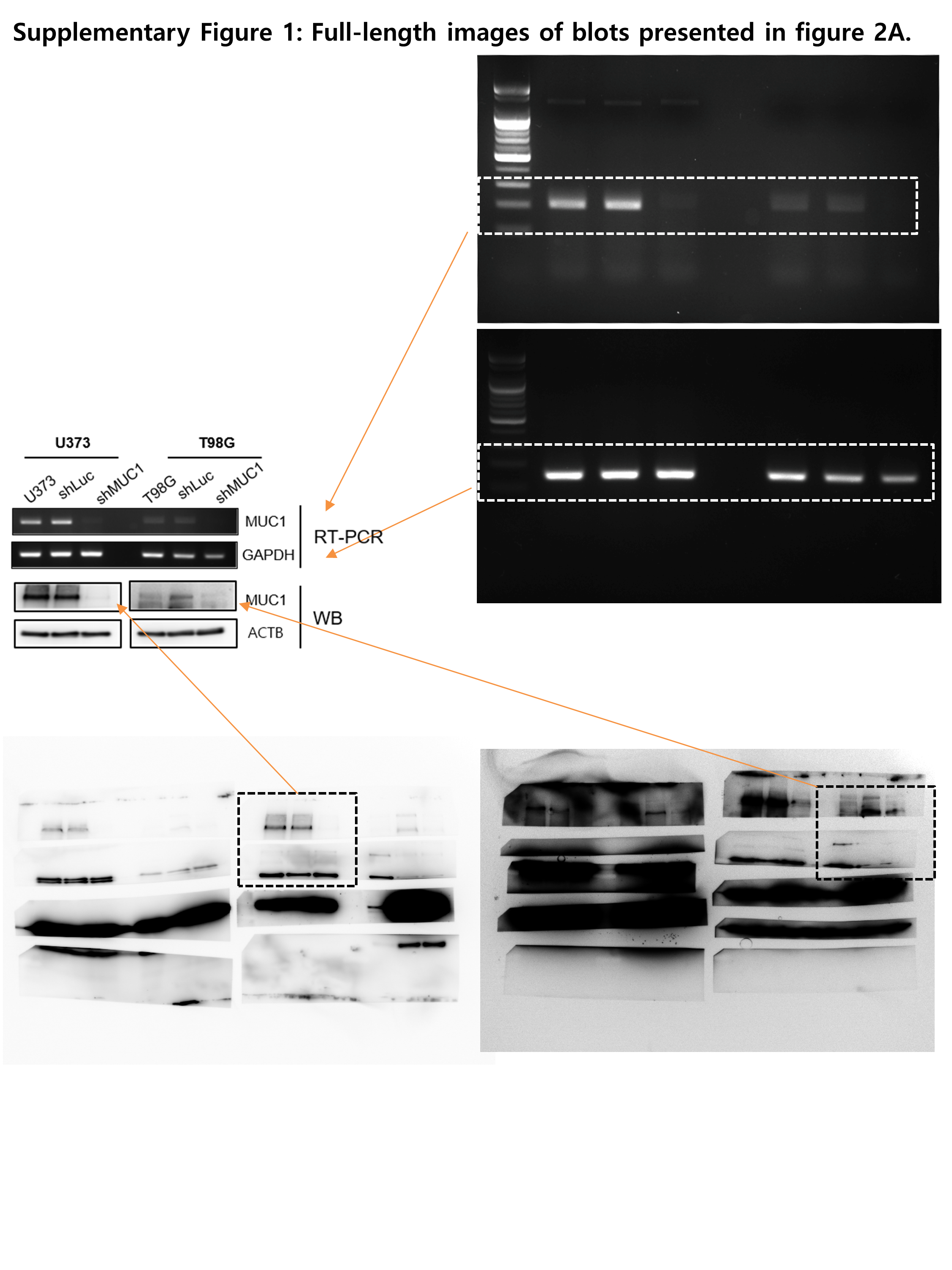


**Supplementary Figure 2**: Full-length images of blots presented in figure 3C. Chemiluminescent signals were detected by exposure of the membranes in a Bio-Ras ChemiDoc system and the protein bands shown in Figure 3C are indicated by dotted boxes.


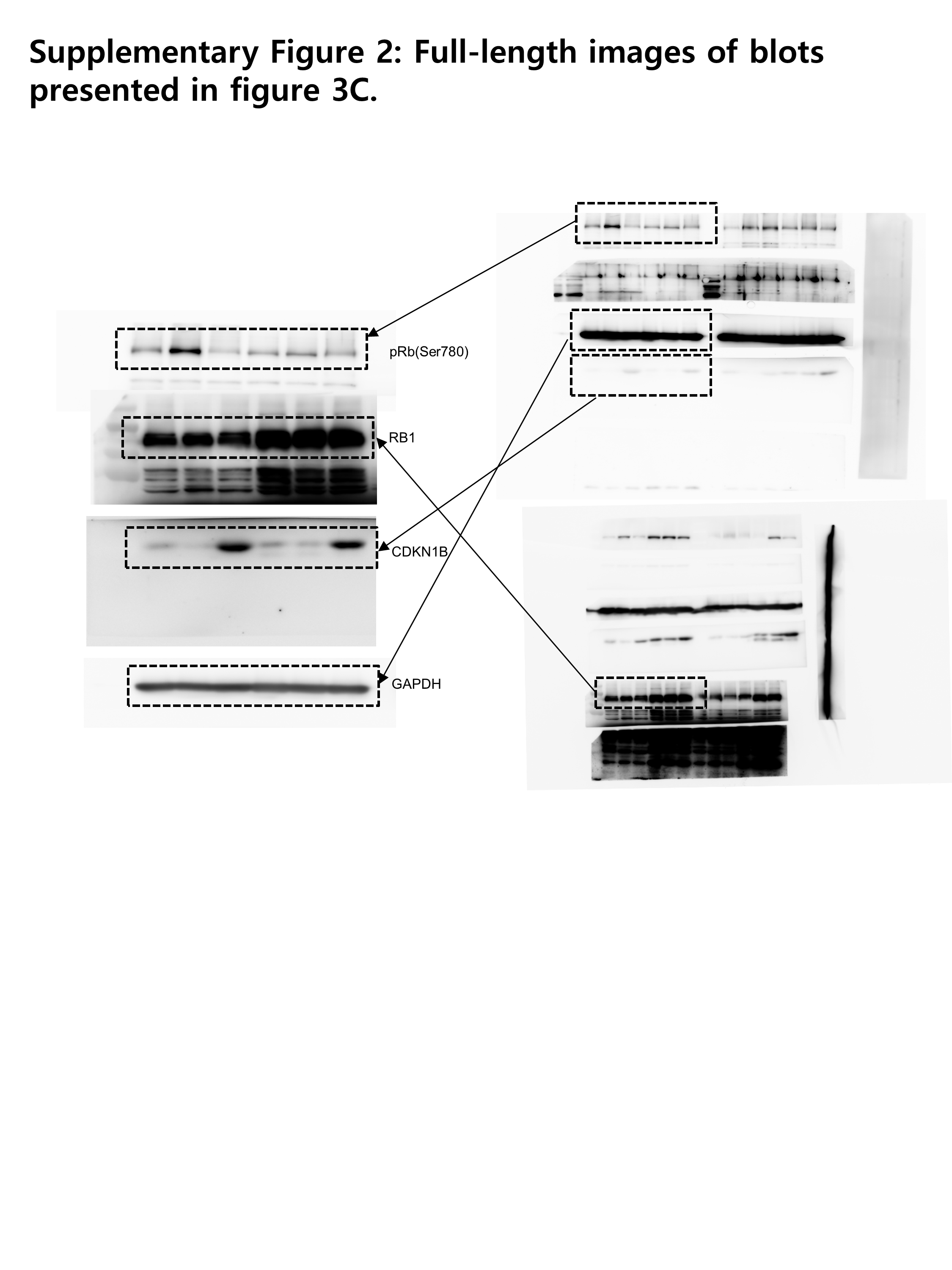


**Supplementary Figure 3:** Full-length images of blots presented in figure 5. Chemiluminescent signals were detected by exposure of the membranes in a Bio-Ras ChemiDoc system and the data shown in Figure 5C are indicated by dotted boxes.


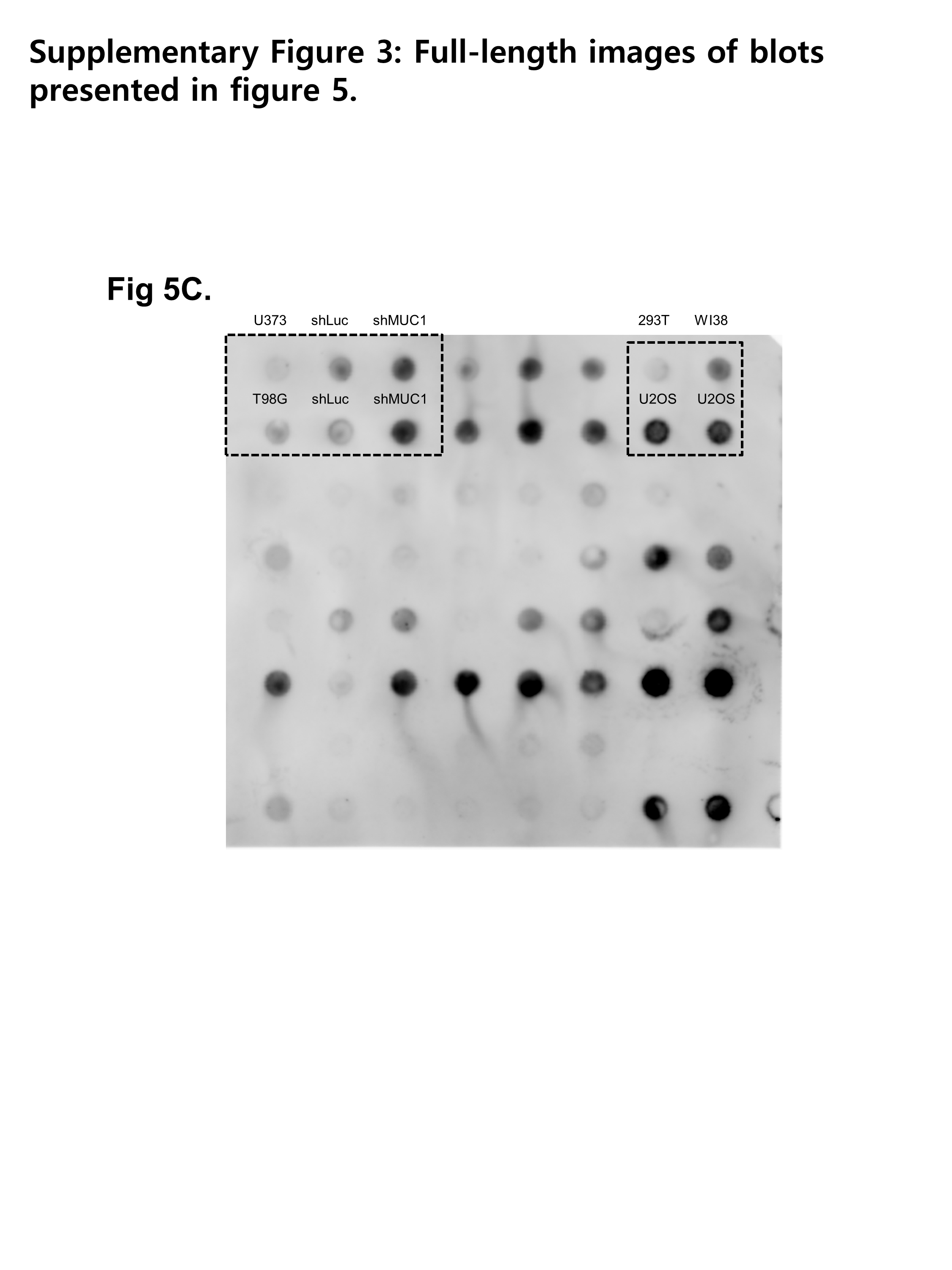


**Supplementary Figure 4:** Annexin V apoptosis assay. Control cells and MUC1 knockdown cells were stained by APC Annexin V Apoptosis Detection Kit with 7-AAD, and apoptosis was analyzed by flow cytometry. Cells were divided into four sections (Q1-Q4): Q1 (AnnexinV-APC-/7AAD+) is representative of mechanical error; Q2 (AnnexinV-APC+/7AAD+) is representative of late apoptotic or necrotic cells; Q3 (AnnexinV-APC-/7AAD−) is representative of living cells; Q4 ((AnnexinV-APC+/7AAD‐) is representative of early apoptotic cells. No statistical difference was noted between control cells and MUC1 knockdown cells.


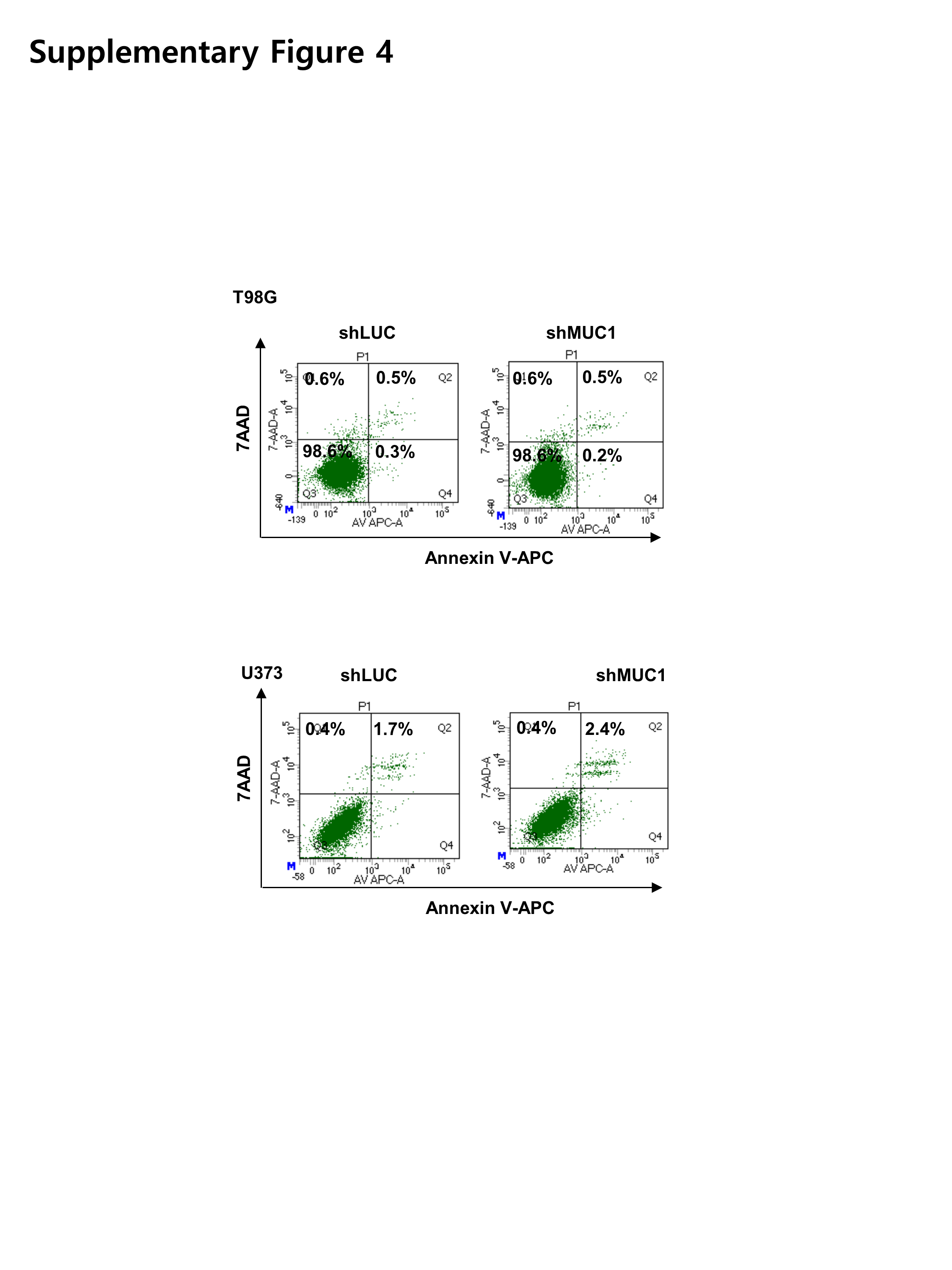

Supplement: Supplementary file 1 — Supplementary Information. [file 41598_2020_75457_MOESM1_ESM.docx]
